# Does the Creation of Food Safety Demonstration Cities Promote Agricultural Development? Evidence from China

**DOI:** 10.3390/ijerph192416961

**Published:** 2022-12-16

**Authors:** Jie Lv, Lu Huang, Xiaoting Li

**Affiliations:** 1 School of Economics, Shandong Technology and Business University, Yantai 264003, China; 2 School of Economics, Shandong University of Finance and Economics, Jinan 250014, China; 3 School of Business, Shandong Jianzhu University, Jinan 250100, China

**Keywords:** food safety demonstration city, agricultural development, difference-in-differences model, China

## Abstract

The creation of food safety demonstration cities (CFSDC) is among the key measures the Chinese government implemented to address the intensifying food safety problem. While effectively managing food safety issues, whether this measure will have an impact on agricultural development in China remains unknown. In this paper, based on panel data from 277 cities in China from 2011 to 2019, the impact of the construction of food safety demonstration cities on agricultural development is empirically examined using the difference-in-differences (DID) model. The results show that the CFSDC significantly improves the level of agricultural development, which still holds after various robustness tests. Analysis of the underlying mechanism indicates that the CFSDC promotes urban agricultural development by accelerating both technological innovation and industrial restructuring. The results of heterogeneity analysis show that the policy effects of the CFSDC exhibit significant heterogeneous characteristics depending on city size, city location, and city administrative level. The findings contribute to the understanding of the relationship between policy pilot projects and agricultural development and provide empirical support for promoting the governance of food safety issues and agricultural development.

## 1. Introduction

Most countries in the world, including China, have been affected by food safety issues in recent decades [1]. From an economic perspective, the existence of information asymmetry may be an important reason for the frequent occurrence of problems in the food market [2]. Food safety incidents have endangered people’s health while also causing the public to question the credibility of the government [3,4]. Therefore, there is an urgent need to address food safety issues, with the goal to enhance public well-being. In this context, the government is increasingly emphasizing the governance of food safety issues [5]. As the ultimate source of food safety problems, the increasing level of agricultural development provides a fundamental guarantee for solving food safety problems [6].

Agriculture occupies a central place in the economic composition of any country [7]. In recent years, China’s agricultural development has gradually entered a new normal, and the structure of the agricultural industry has been continuously improved. The agricultural production layout has been increasingly enhanced, and perceptible development has been achieved [8]. However, in the process of China’s agricultural development, the excessive use of agricultural chemical inputs has resulted in serious agricultural non-point-source pollution; moreover, the lack of agricultural machinery power and highly qualified agricultural technical personnel has resulted in a low agricultural input–output efficiency for a long time. Under the currently intensifying food-safety pressure, agricultural development has been seriously affected [9]. In particular, the agricultural economy grew only 3.1% in 2019, which signifies a record low since 2004. At the same time, it has been shown that there is still much room for improvement in China’s agricultural development, especially regarding precision agriculture development [10].

Promoting the efficient use of agricultural land has become a global challenge [11]. To cope with emerging problems of agricultural development under the new economic normal and to accelerate the process of agricultural and rural modernization, in 2021, the Chinese government released the “Opinions of the Central Committee of the Communist Party of China and the State Council on Comprehensively Promoting Rural Revitalization and Accelerating Agricultural and Rural Modernization”. These opinions clearly stated the importance of the task to strengthen the basic position of agriculture and enhance the quality, efficiency, and competitiveness of agriculture in the “14th Five-Year Plan” period. China’s emphasis on agriculture has been raised to an unprecedented level. The government has realized that the vulnerability associated with agricultural development will affect the health and life of the public in the near future [12].

Policy practice creation provides observable first-hand policy experience for related research in an attempt to transition from theory to practice [13,14]. In recent years, numerous scholars have focused on the impact of policy practice on agricultural development [15,16,17,18]. As a policy prescription with the goal to enhance agricultural development in developing countries, the impact of agricultural land titling on agricultural development has been studied from the perspective of property rights systems in breaking up small-scale operations [19]. However, it has been suggested that the agricultural land rights confirmation system inhibited farmland transfer [20]. The implementation effect of precision agriculture policies has also been studied. Research showed that although precision agriculture policies are indeed effective in China’s agricultural development, the adoption rate of precision agriculture is low. Among the important reasons is that farmers have different views on agricultural policies and intentions to implement these. Measures such as increasing policy subsidies and agricultural loans would be beneficial for the implementation of this policy [21]. Going one step further, scholars have studied the implementation effect of digital policies in the agricultural sector. Research showed that although digital policies are important for sustainable agricultural development, how such digital policies manage to enhance agricultural production efficiency and reduce undesired outputs remains unclear [22]. Similarly, research on the implementation effect of the agricultural policy on genetically modified foods showed that although agriculture using genetically modified crops is regarded as an important way to increase the output of the agricultural industry; it remains unknown whether this policy can promote the sustainable development of agriculture [23]. Additionally, it has been suggested that the effect of certain agricultural policies depends on specific subjects, some developing countries have not achieved specific policy goals because of a lack of financial resources [24,25]. For example, the African Union should use its influence to reshape the Comprehensive African Agricultural Development Program to promote African agricultural development by increasing investment in public goods [26,27].

Theoretically, the development of agriculture provides additional and alternative food sources for humans, which is an important guarantee for a country’s food security, particularly its grain security [28]. Changes in the demand of food consumption will also naturally affect the development of agriculture, so the two are closely related [29]. In recent years, with their increasingly frequent occurrence, food safety problems have gradually become a “bottleneck” that affects the development of agriculture and food industries of most countries. Thus, food safety problems restrict the strategic restructuring of the rural economy, and food supply chains must be developed more efficiently [30]. Against this background, the Chinese government has implemented many measures, such as reforming the establishment of regulatory agencies and improving the legal system. These measures aim to guide the development of the food industry. While continuous efforts have indeed improved the food safety situation, considerable room for improvement remains [31,32]. The creation of food safety demonstration cities (CFSDC) is also an important measure to improve the current situation of food safety. Through the creation of three batches from 2014 to 2016, there are 67 food safety demonstration cities in China, which have played an important role in solving food safety problems. The CFSDC takes into account the key links such as the planting of edible agricultural products, livestock and poultry breeding, aquaculture, agricultural product processing, and agricultural product circulation. Through the CFSDC, the production and operation level of these key links have been greatly improved, which may have an impact on agricultural development. Then, does the policy practice of establishing “market leading” and “government guided” food safety demonstration cities produce other economic and social benefits while solving food safety problems? Does it also have an impact on agricultural development closely related to food safety?

In light of the above analysis, existing research has not yet addressed the following pertinent questions: Does the CFSDC in China improve agricultural development? What are the impact mechanisms of the policy effect? Are the effects heterogeneous? To address these questions, this paper places the CFSDC and agricultural development in the same research framework. The policy practice of the creation of food safety demonstration cities is regarded as a quasi-natural experiment. The impact of the CFSDC on agricultural development and its mechanism in China are examined using the difference-in-differences (DID) model. The heterogeneity of factors such as city size (megacities, large cities, medium and small cities), city location (eastern region, central region, western region), and city administrative level (high-level cities, low- and middle-level cities) on agricultural development is further discussed.

The contributions of this research are summarized in the following: This paper places the CFSDC and agricultural development in the same research framework, thus studying the impact of the CFSDC on agricultural development. This paper not only enriches research on the evaluation of policy effects of the CFSDC, but also provides new ideas for further explorations on how agricultural development can be improved. The transmission mechanism and heterogeneity of CFSDC affecting agricultural development were also analyzed in this study. This deepened the research on the relationship between food safety policy practice and agricultural development, and provided a reference for developing countries to formulate and implement relevant policies, thus promoting high-quality agricultural development.

## 2. Policy Background and Research Hypothesis

### 2.1. Policy Background

In order to urge the implementation of party and government responsibility for food safety and the “four strictest”, encourage local governments to play a pioneering role, explore food safety governance systems and methods, and demonstrate and drive the improvement of national food safety governance, in July 2014, the Food Safety Committee of the State Council (FSCSC) decided to organize the creation of a national food safety demonstration city, which would be organized and implemented by the Office of the Food Safety Committee of the State Council (OFSCSC). In 2014, the FSCSC announced that 15 cities were included in the first batch of pilot cities, and the creation of food safety demonstration cities was kicked off. In September 2016, the OFSCSC issued the National Food Safety Demonstration City Evaluation and Management Measures (Provisional) and the National Food Safety Demonstration City Standard (2017 Edition).

The creation of food safety demonstration cities is divided into the following four parts: pilot creation, preliminary evaluation and recommendation at the provincial level, public evaluation at the national level, and licensing and naming. The preliminary evaluation and recommendation at the provincial level are directed by the provincial food safety committee of the province where the city is located. Two methods are used: entrusting a third-party evaluation or evaluation by the provincial food safety committee and relevant departments. However, food safety satisfaction studies must be completed by an independent third-party agency. For cities participating in the creation of food safety demonstration cities, in principle, the creation cycle takes at least two years. During the creation cycle, it is necessary for participating cities to focus on optimizing and adjusting issues such as “source governance”, “whole process supervision”, “corporate responsibility”, and “social co-governance”. Cities that pass the publicity evaluation at the national level will be awarded the title of “National Food Safety Demonstration City”.

To date, the FSCSC has successively established three batches of national food safety demonstration cities in 2014, 2015, and 2016, increasing the number of established cities to 67, covering all provinces in China. In June 2017, the first batch of 15 cities that participated in the creation of national food safety demonstration cities passed inspection and were awarded the title. Since the creation of the first food safety demonstration city, food safety issues have been effectively managed in all regions, the food industry has been operating well, and the people’s satisfaction with food safety has increased year by year. Through the food safety demonstration, China’s food safety governance level has been greatly improved. To be specific, through the CFSDC, China has formed important experience in the following aspects: the food safety situation continues to be good, the party and the government have implemented their responsibilities together, the source of food safety has been effectively managed, the whole process of supervision has been strictly implemented, illegal and criminal acts have been severely hit, corporate responsibility has been fully implemented, and the pattern of social co governance has basically taken shape. This has provided a solid guarantee for food safety in China’s economic development.

### 2.2. Research Hypothesis

**Figure 1:**
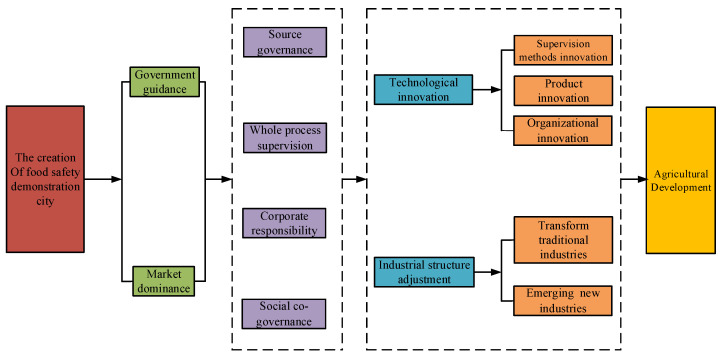
Mechanism of effects of the creation of food safety cities on agricultural development.

In theory, the most direct impact of the CFSDC is the improvement of the level of urban food safety. However, while food safety demonstration cities are required to provide multiple guarantees of institutions, personnel, financial resources, and authority, the scope of the food safety model city creation policy mainly revolves around agriculture. Pilot cities should impact the development of agriculture in addition to their original role of improving food safety. Existing research showed that technological innovation and industrial structure adjustment are dual pillars needed to transform the mode of economic development and realize sustainable regional development [33]. Based on the theory of institutional change and agricultural development, this section summarizes the impact mechanisms of the CFSDC on agricultural development (as shown in Figure 1).

The National Food Safety Demonstration City Standard (2017 Edition) proposed that cities participating in the creation of food safety demonstration cities should strictly implement the source supervision of agricultural products and improve the inspection and testing capabilities of agricultural products. Among the prerequisites for the high-quality development of agriculture lies in accelerating technological innovation and applying smart agriculture [34]. The CFSDC has the following impacts on agricultural technological innovation: On the one hand, the CFSDC has promoted the innovation of supervision methods of agricultural production. Cities designated to become food safety demonstration cities should equip front-line supervision and law enforcement personnel with information-based terminal equipment to improve their mobile supervision and law enforcement capabilities. In addition, food safety maps, agricultural input management ledgers, and traceable quick response codes should be used. The use of innovative and information-based means has improved the food safety supervision ability. The innovation of supervision directly affects the production and operation activities of agricultural enterprises, thus affecting the development of agriculture.

On the other hand, the CFSDC promotes production innovation in agricultural enterprises. The CFSDC promotes the application of informatization and intelligent technology for the production and operation processes of agricultural enterprises, thereby enhancing the added value of agricultural products.

Finally, the CFSDC promotes organizational innovation in agribusinesses. The CFSDC has introduced clearer requirements for the operation and management of agricultural enterprises. This clarification has led to the development of an organizational management model for agricultural enterprises towards scientific management, information management, and network management. Based on the above-mentioned content, the CFSDC has improved the level of technological innovation in participating cities and has thus influenced agricultural development.

**Hypothesis** **1 (H1).** *The creation of food safety demonstration cities (CFSDC) promotes agricultural development by enhancing the city’s technological innovation level.*

Cities participating in the creation of food safety demonstration cities can draw on preferential policies in several areas, including institutions, personnel, financial resources, and authority. Consequently, the transformation and upgrading of urban industrial structures can be promoted, resulting in policy effects that improve agricultural production. On the one hand, the CFSDC has promoted both the transformation and upgrading of traditional agricultural production. With the advancement of the policy for establishing food safety demonstration cities, the traditional high-pollution and low-value-added agricultural production and operation model has become unsustainable. Considering that the public demand for high-quality agricultural products is increasing, the National Food Safety Demonstration City Standard (2017 Edition) has included specific provisions on the use of agricultural and veterinary drugs, the requirements for pesticide residue standards, and the treatment of dead livestock and poultry. This prompts enterprises that are engaged in traditional agricultural production to choose cleaner production technologies and achieve green agricultural production. However, certain high-polluting agricultural enterprises will actively move out of pilot cities driven by the “pollution shelter” effect, thus driving the development of regional transformation and the upgrading of traditional agricultural production.

On the other hand, the CFSDC led to the emergence and development of new industries. In addition to promoting traditional high-pollution, low value-added agricultural production and operation models, the CFSDC is also an effort to build up the whole agricultural industry chain and value chain, and promote the high-quality transformation of agriculture. Cities participating in this policy pilot will strive for project, technical, and financial support to build advantageous and characteristic industrial cluster construction projects in the region. Further, they will also develop characteristic agricultural product production based on market demand to form “cooperatives + farmers” and “cooperatives + logistics + e-commerce” constructs. A variety of new production and operation modes, and guidance for agricultural enterprises to move from decentralized development to centralized development, promoted the extension of the industry before and after the pilot. Further, the goal of “connecting the market above and connecting the elements below” is achieved. Based on the above-mentioned content, the CFSDC has promoted the adjustment of the industrial structure of the city and has thus affected the development of agriculture.

**Hypothesis** **2 (H2).** *The creation of food safety demonstration cities (CFSDC) promotes agricultural development by accelerating the city’s industrial restructuring.*

## 3. Materials and Methods

### 3.1. Model Construction

In reference to existing research [35], to effectively identify the impact the CFSDC has on agricultural development, this paper examines the causal effects of policy pilots on agricultural development by constructing a multi-period DID model. This model was set up as follows:

(1) $$Y_{it}=\beta_{0}+\beta_{1}Treat_{i}\cdot Time_{t}+\beta_{2}X_{it}+\mu_{i}+\gamma_{t}+\varepsilon_{it}$$

In Equation (1), i represents the year; t represents the city; $Y_{it}$ is the explained variable, representing the agricultural development level of each city in different years; $Treat_{i}$ is the dummy variable between groups: when the city is determined to be a food safety demonstration city, the value is 1, otherwise, the value is 0; $Time_{t}$ is a time dummy variable, which takes a value of 0 before the city is determined to be a food safety demonstration creation city, and a value of 1 afterwards; the estimation coefficient of $Treat_{i}\cdot Time_{t}$ is the focus of this paper, which reflects the net effect of the policy pilot on agricultural development; $X_{it}$ is a series of control variables; $\mu_{i}$, $\gamma_{t}$ and $\varepsilon_{it}$ represent the region fixed effect, the time fixed effect, and the random disturbance term, respectively.

### 3.2. Variable Selection

#### 3.2.1. Explained Variable

In this research, the explained variable is the agricultural development level (ADL), which is measured by the ratio of the output value of the primary industry to the gross domestic product (GDP). There were three considerations when selecting this indicator. First, the most direct advantage of this approach is that the data at the level of prefecture-level cities in China can be completely obtained while ensuring the relevance. Second, agricultural products are directly derived from agricultural production activities, which belongs to the primary industry. Although food products produced through industrial processing belong to the secondary industry, most of the raw materials they need come from the primary industry. Agricultural production technology, agricultural machinery power, high-quality labor force, agricultural capital and other elements are intermediate variables in the process of agricultural development. The final outcome variables generated from their distribution and use will be reflected in the form of the output value of the primary industry. Therefore, the output value of the primary industry can well measure the agricultural production and operation results related to food. The output value of the primary industry can not only reflect the level of agricultural development in terms of quantity, but also reflect the quality of agricultural development in terms of quality. Third, the primary industry includes traditional agriculture, forestry, animal husbandry, and fishery, which covers a wider range than the agricultural output value index and better matches the coverage of food safety demonstration cities [36]. The proportion of the output value of the primary industry in GDP is used as a reflection of the relative level of agricultural development, and the analysis of the agricultural output value is introduced in the robustness test.

#### 3.2.2. Explanatory Variable

The core explanatory variable is the food safety demonstration city creation policy (Treat·Time), which is measured by the interaction term between the dummy variable of groups and time. After processing, if a city creates or has created a food safety demonstration city in a certain year, it will be set to 1, otherwise it will be set to 0.

#### 3.2.3. Control Variables

The control variables are: (1) Cultivated land area (CLA), which is the factor most directly affecting agricultural development. The CLA forms the premise and basis for the expansion of the agricultural development scale. In this paper, the logarithm of the urban effective irrigation area is used as measurement index of CLA [37]. (2) The use of agricultural machinery (UAM) is among the important symbols of the development of modern agriculture, and it is also among the ways for improving both agricultural production efficiency and agricultural production conditions. The logarithm of the total power of urban agricultural machinery is used as a measure of agricultural machinery use [37]. (3) The use of agricultural plastic film (UAP) plays an important role at the sowing stage of agricultural production for heat preservation and moisture retention, which is essential for improving agricultural production. In this paper, the logarithm of the using area of agricultural plastic film is used to measure the UAP [38]. (4) Improving the educational development level (EDL) can improve the skill of laborers and foster agricultural human capital. To assess the role of accumulation, this paper uses the logarithm of the number of urban college students as a measure of the level of educational development [39]. (5) The degree of opening to the outside world (DOO) is also measured, because the opening of a city to the outside world results in the influx of both advanced agricultural production technology and agricultural management experience. This will also accelerate the development of the manufacturing and service industries in the region, which directly affects the intensity of agricultural production and forms an important factor in the development of urban agriculture [40].

Based on the list of food safety demonstration cities announced by the FSCSC, for this study, 277 Chinese cities were selected and data were collected from 2011 to 2019. The experimental group contained 65 cities (data on the Yangling demonstration area are missing, which was therefore excluded; Hancheng was managed by Weinan City, and was therefore also excluded). The rest of the cities were used as control group. The relevant data were mainly obtained from the China Urban Statistical Yearbooks, and missing data were obtained by querying the relevant statistical yearbook of the province.

## 4. Results

### 4.1. Parallel Trend Test

**Figure 2:**
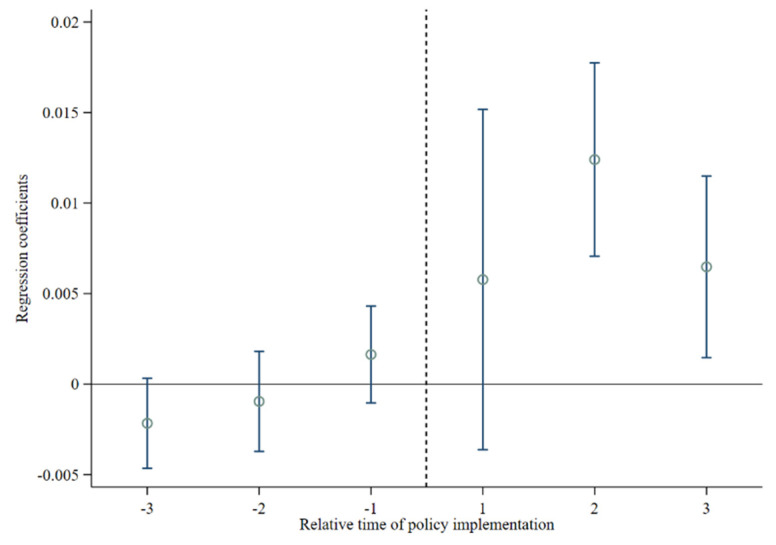
Parallel trend test results. Note: the horizontal axis represents the relative time point of food safety demonstration city creation. The vertical axis represents the regression coefficient of food safety demonstration city creation on agricultural development under the 95% confidence interval.

When using the DID method to evaluate policy effects, the basic assumption of a parallel trend must be met [41]. This means that if the policy of creating food safety demonstration cities is not implemented, after controlling for a series of observable factors, the agricultural development trends of the experimental group and the control group should remain unchanged. In reference to existing research [42], this paper adopts the event study method to conduct parallel trend test. The results are shown in Figure 2.

Before the creation of food safety demonstration cities, the regression coefficients of food safety demonstration cities on agricultural development were mostly negative and failed to pass the significance test. This result indicates that there was no obvious difference in agricultural development between experimental and control group. Therefore, the basic assumption of parallel trends is satisfied. The promotion effect of their on agricultural development began to appear in the second year after the creation of food safety demonstration cities. This shows that the policy impact of the CFSDC on agricultural development has a certain time lag. In addition, Figure 2 shows that after the creation of food safety demonstration cities, the regression coefficient of food safety demonstration cities showed a trend of first increasing and then decreasing. The possible reason is that the technological innovation effect and industrial structure adjustment effect (both of which are part of the CFSDC) can promote the improvement of ADL in the short term. However, in the long term, the policy effect is not sufficiently dynamic, and agricultural development must be adjusted at a deeper level and from within the industry.

### 4.2. Baseline Regression

Table 1 reports the estimation results of the baseline regression model. Model 1 only uses the food safety demonstration city policy as an explanatory variable, while other control variables are excluded. Models 2 to 6 show the estimation results after sequentially including CLA, the UAM, the UAP, improving the EDL, and the DOO.

This paper focuses on the regression coefficients of the policy variables for the CFSDC. The regression results of Models 1 to 6 show that the regression coefficients are all positive. This shows that the CFSDC has significantly promoted the agricultural development of these cities. The regression results based on Model 6 show that the CFSDC drives the growth of agriculture by 0.77%. This means that the CFSDC can not only rectify the development order of the food industry, but also increase both agricultural production and income.

The regression results of the control variables show that both the positive and negative directions of coefficients match the relevant economic theories. On the one hand, from the perspective of the factors of production affecting agricultural development, the regression coefficients of the variables of CLA, total power of agricultural machinery, and agricultural plastic film usage are all positive. This indicates that the improvement of these factors of production can drive agricultural development. On the other hand, judging from the significance of the regression coefficients, the regression coefficients of the factors of production failed to pass the significance test. This indicates that serious problems of low input–output efficiency may exist in the current agricultural development, and an agricultural production model is urgently needed. The current model must be advanced from the rough production mode to the refined production mode. Although the regression coefficient of the EDL is positive, it is not significant, which further shows that the current agricultural development urgently needs to introduce agricultural talents with professional and technical capabilities. The regression coefficient of the DOO is significantly negative. This indicates that the opening of cities to the outside world not only introduces advanced agricultural production technology and agricultural management experience, it also accelerates the development of the manufacturing and service industries in the region. This development directly affects the layout of agricultural production in the region, causing a shift in the focus of development in the region to other industries and sectors.

### 4.3. Robustness Test

#### 4.3.1. PSM-DID Test

Theoretically, if the policy shock for the CFSDC is strictly exogenous, the causal relationship can be identified through the DID method. However, areas identified as food safety demonstration cities are not generated by random exogenous shocks during the design of the policy. They are influenced by many factors such as the economic and social development of the area, the development of the food industry, and the strength of food safety supervision [43]. Therefore, propensity score matching difference-in-differences (PSM-DID) was used to exclude possible exogeneity. Based on the basic principle of the PSM-DID test, in this section, three matching methods are used (i.e., kernel matching, radius matching and nearest neighbor matching) to match the samples set up as food safety demonstration cities to the control group. Then, the DID method is used to identify the net effect of food safety demonstration cities on agricultural development.

The results are reported in Table 2. The results show that the regression coefficients are all significantly positive. This is consistent with the regression coefficients of policy variables in the benchmark regression results, indicating that the CFSDC promotes agricultural development, and that the relationship is causal.

#### 4.3.2. Placebo Test

**Figure 3:**
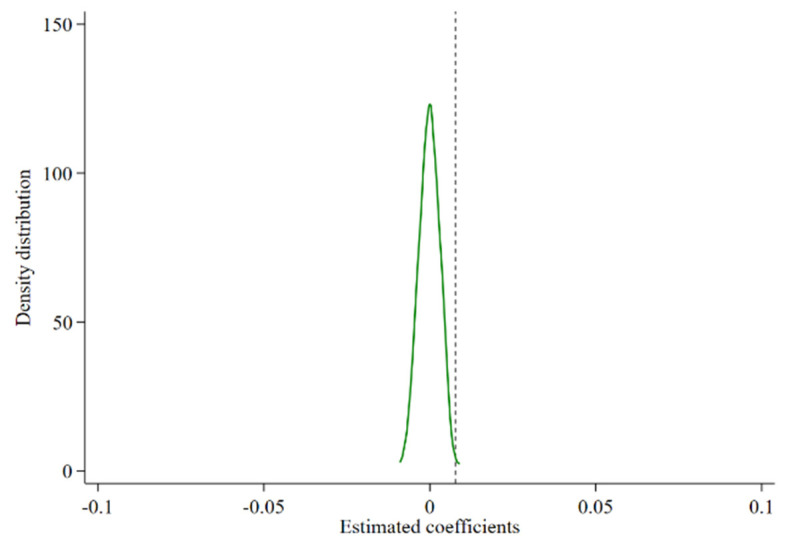
Placebo test results.

In order to ensure that the establishment of food safety cities has a strong robustness on the impact of agricultural development, and to test whether the policy degree of food safety city establishment is affected by regional characteristics and random factors, this paper constructs a placebo test by randomly generating an experimental group that implements the policy of food safety city establishment. 65 cities were randomly allocated to a new experimental group [44]. The remaining cities formed the control group, and 500 regressions were conducted for the quasi-regression model. The regression results show that the mean value of the regression coefficient of the policy variable after random treatment is 0, and fails the significance test. This result indicates that the causal effect of the CFSDC to promote agricultural development does not originate from urban characteristics or unobservable factors. Figure 3 shows the Kernel density estimation graph of the estimated coefficients of food safety demonstration cities based on the results of these 500 random iterations. The dashed line indicates the coefficient of explanatory variable in the baseline regression results. The regression coefficients after random processing are distributed around 0, indicating strong robustness of the results of the previous analysis.

#### 4.3.3. Other Robustness Tests

For the first further robustness test, the control variable was lagged in one period. Considering the potential endogeneity issue and for reducing the inverse effect between the CFSDC and the selected variables, all control variables are lagged by one period. Moreover, both the control variables and policy variables are regressed with the explanatory variables after the lag. The results are shown in Column 2 of Table 3. The re-regression results show that after controlling for control variables, the regression coefficients of policy variables are still significantly positive and the magnitude of the coefficients does not significantly differ from the baseline regression results. This implies that the findings of the previous study are robust.

The second further robustness test used abbreviated processing. To reduce the effect extreme outliers have on the results of this research, all variables are winsorized by 0.5%. The results after re-regression are shown in Column 3 of Table 3. The regression results show that the regression coefficient of the policy variable after the abbreviated treatment is 0.0074, passing the significance test at the 1% level. There is no significant change in the coefficient size and significance level compared to the regression coefficient of policy variables in the benchmark regression results, which further corroborates the robustness of the benchmark regression results.

In the third further robustness test, the sample interval is adjusted. The robustness of the previous study results is analyzed by changing the time span of the study. Specifically, the study data from 2011 and 2019 are excluded, and only the study data from 2012 to 2018 are retained. The re-regression results are shown in Column 4 of Table 3. The regression results show that the regression coefficient of the policy variable remains significantly positive, with no significant change in the magnitude or significance level of the coefficients. This further corroborates the robustness of the previous study results.

In the fourth further robustness test, the explained variable is replaced. So far, the explained variable in the benchmark regression can broadly be regarded as the ADL. In this test, the ADL is replaced with the agricultural output value, i.e., the ADL in the narrow sense. The re-regression results show that the regression coefficient of policy variables remains significantly positive, and both the magnitude and significance level of coefficients remain unchanged. This further confirms the robustness of the results so far.

### 4.4. Mechanism Analysis

Baseline regression and a series of robustness tests confirm that the CFSDC significantly promotes agricultural development. The remaining question is how the creation of food safety model cities could impact agricultural development? In the analysis so far, technological innovation and industrial structure adjustment played an intermediary role in the CFSDC to promote agricultural development. In this section, the mediating effect is tested.

#### 4.4.1. Mediating Effect Model Construction

In reference to existing research [45], the following steps were taken to set the mediating effect model: First, the level of agricultural development is taken as the explained variable, and the CFSDC is taken as the core explanatory variables, which are regressed (i.e., the benchmark regression). If the regression coefficient of the CFSDC is significantly positive, the CFSDC promotes agricultural development. Second, technological innovation and industrial structure adjustment are used as explained variables, and are incorporated into the regression model to examine the impact of the CFSDC on technological innovation and industrial structure adjustment, respectively. If the regression coefficient of the CFSDC is significantly positive, the CFSDC promotes industrial structure adjustment and technological innovation in cities. Third, technological innovation and industrial structure adjustment are taken as core explanatory variables for the regression model to examine the impact of technological innovation and industrial structure adjustment on agricultural development, respectively. If the regression coefficients of technological innovation and industrial structure adjustment are both significantly positive, technological innovation and industrial structure adjustment promote the agricultural development of the city. Fourth, the CFSDC and the mediating variables are incorporated into the regression model and their impact on agricultural development is analyzed. Following these steps, the mediation effect model can be written as follows:

(2) $$Y_{it}=\beta_{0}+\beta_{1}Treat_{i}\cdot Time_{t}+\beta_{2}X_{it}+\mu_{i}+\gamma_{t}+\varepsilon_{it}$$

(3) $$Med_{it}=\alpha_{0}+\alpha_{1}Treat_{i}\cdot Time_{t}+\alpha_{2}X_{it}+\mu_{i}+\gamma_{t}+\varepsilon_{it}$$

(4) $$Y_{it}=\delta_{0}+\delta_{1}Med_{it}+\delta_{2}X_{it}+\mu_{i}+\gamma_{t}+\varepsilon_{it}$$

(5) $$Y_{it}=\varphi_{0}+\varphi_{1}Treat_{i}\cdot Time_{t}+\varphi_{2}Med_{it}+\varphi_{3}X_{it}+\mu_{i}+\gamma_{t}+\varepsilon_{it}$$

The factors of the above equations are the same as those in Equation (1). It should be noted that $Med_{it}$ in Equation (3) represents the state of technological innovation (industrial structure adjustment), which is measured by the logarithm of the number of invention patents per capita in the city in a certain year [46]. As a measure of technological innovation, the number of invention patents per capita can well reflect the overall innovation capacity of the region. The impact of the improvement of the overall innovation capability on agricultural development is shown in three aspects: First, regulators have the ability to use more advanced supervision equipment and use more advanced supervision means, which will promote the realization of smart agricultural supervision; Second, the application of information and intelligent technology in the production and operation process of agricultural enterprises will help to enhance the added value of agricultural products, which will promote the innovative development of agriculture; Third, the innovative development of information management, network management and other models will help to improve the modern management level of agricultural enterprises, which will promote the standardized development of agriculture.

The ratio of the added value of the tertiary industry to the secondary industry is used as a measure of industrial structure adjustment [47]. It can not only reflect the overall industrial layout of the region, but also reflect the overall development space of agriculture. The impact of the improvement of this indicator on agricultural development is shown in two aspects: first, it means that the economic development has entered a higher stage, and the traditional agricultural production and operation model with high pollution and low added value will be difficult to continue, and the development of traditional agriculture needs transformation and upgrading; second, high-quality consumer demand will stimulate the emergence and development of emerging industries. It is imperative to carry out characteristic agricultural production around market demand and realize the agricultural production mode of “connecting the upper market and the lower factors”.

#### 4.4.2. Mediating Effects of Technological Innovation

In this section, the mediating effect is tested by following the steps in Equations (2)–(5) using technological innovation as mediating variable. Table 4 reports the mediating effect test results of technological innovation after controlling the control variables. Column 2 of Table 4 reports the benchmark regression results. Column 3 of Table 4 reports the regression results with technological innovation as explanatory variable. The results show that the regression coefficient of policy variables is positive, indicating that the CFSDC has significantly promoted technological innovation in pilot cities. Column 3 of Table 4 reports the regression results when agricultural development is used as explained variable and technological innovation as core explanatory variable. The regression coefficient of technological innovation is 0.0019, which passes the significance test at the 1% level, showing that technological innovation has significantly boosted urban agricultural development. Column 5 of Table 4 reports the regression results when taking agricultural development as explained variable and the CFSDC as core explanatory variable, while also incorporating technological innovation into the regression model. The regression coefficient of the policy variable is 0.0055, showing that the coefficient size and significance level have decreased compared with the baseline regression results. This indicates that the CFSDC can promote agricultural development. The regression coefficient of technological innovation is 0.0017. This result indicates that the CFSDC promotes urban agricultural development through technological innovation.

#### 4.4.3. Mediating Effects of Industrial Structure Adjustment

In this section, industrial structure adjustment is used as mediating variable to test the mediating effect. Table 5 reports the results of the mediating effect test of the industrial structure adjustment after controlling for control variables. Column 2 of Table 5 reports the benchmark regression results, and Column 3 reports the regression results with industrial structure adjustment as explained variable. The results show that the regression coefficient of the policy variable is significantly positive, indicating that the CFSDC has significantly promoted the industrial structure adjustment of pilot cities. Column 4 of Table 5 reports the regression results with agricultural development as explained variable and industrial structure adjustment as core explanatory variable. The adjustment of the industrial structure has significantly promoted the agricultural development of pilot cities. Column 5 of Table 5 reports the regression results when using agricultural development as explained variable and the CFSDC as core explanatory variable, while adding the adjustment of industrial structure into the regression model. The regression coefficient of the policy variable is 0.0064, which is significant and positive, and passes the significance test at the 1% level. The coefficient is lower than the 0.0077 of the benchmark regression result, which further demonstrates that the CFSDC can promote agricultural development. The regression coefficient of industrial structure adjustment is 0.0237 and passes the significance test at the 1% level. This indicates that the CFSDC promotes urban agricultural development through adjusting the industrial structure.

### 4.5. Heterogeneity Analysis

In theory, the implementation of a policy pilot activity should have the same or similar effects for all pilot cities; however, in reality, the policy pilot has had different and even opposite effects in each city, depending on factors such as city size, city location, and city level. The heterogeneous impact of the CFSDC on agricultural development is analyzed from the perspectives of city scale, city location, and city level.

#### 4.5.1. Heterogeneity Analysis of Urban Scale

In reference to the “Notice on Adjusting the Criteria for Urban Scale Division” issued by the State Council in 2014, the 277 studied cities are classified into three types of cities based on their population size: megacities, large cities, and small-medium-sized cities. Cross-multiplication of city size dummy variables with policy variables is conducted to reflect the impact of city size on agricultural development. Megacities are cities with a permanent population of 5 million or more at the end of the year; large cities are cities with a permanent population between 1 million and 5 million at the end of the year (including 1 million, but excluding 5 million); small- and medium-sized cities are cities with a permanent population of less than 1 million at the end of the year. The results of the impact of urban size heterogeneity on ADL are shown in Table 6.

The regression coefficients of policy variables are significantly positive in all three city types, indicating that the CFSDC has indeed promoted agricultural development. The regression coefficient of the policy variable of the large city sample is consistent with the baseline regression. However, the regression coefficient of the policy variable of the mega-city sample is smaller than the regression coefficient of the policy variable in the benchmark regression results. The policy variable regression coefficient of the medium and small city sample exceeds that of the benchmark regression results, showing that the CFSDC has had a varying effect on cities of different scales. A possible reason is that the mega-city sample has a low policy effect of the pilot policy because of the considerable resources available for its development. This moves the focus of urban development towards modern services, leaving relatively little resources for agricultural development. Moreover, as the economic and social development of mega-cities is at the leading edge, it is far more difficult for these cities to overcome limitations and improve further compared to other types of cities. The sample of large cities reflects the average effect of the CFSDC. Because of the influence of the industrial structure, medium and small cities pay relatively high attention to agriculture and have consequently received more attention when implementing the policy of establishing food safety demonstration cities. Therefore, this policy may have a greater impact on agricultural development in medium and small cities.

#### 4.5.2. Heterogeneity Analysis of Urban Location

Numerous studies have shown that China’s economic and social development is characterized by significant regional differences, where different locations imply differences in the distribution of both cities and urban governance capacity [48]. Based on this, it is assumed that the impact of the CFSDC on agricultural development may differ depending on differences in the area the cities are located in. In reference to the division criteria of the National Bureau of Statistics, the 31 provinces of the sample are divided into the three regions of eastern, central, and western. The 277 cities are further subdivided into three samples of eastern, central, and western cities according to the provinces where they are located. A dummy variable of urban location is constructed, and its influence on agricultural development is assessed by multiplying the dummy variable of urban location with the policy variable. The sample of the eastern region contains 100 cities, the sample of the central region contains 101 cities, and the sample of the western region contains 76 cities.

The results of the impact of urban location heterogeneity on agricultural development from Models 1 to 3 are shown in Table 7. Judging from the positive and negative directions of the regression coefficients of policy variables, the regression coefficients of policy variables are significantly positive in the eastern region. This indicates that the CFSDC has significantly promoted the agricultural development in the eastern region. A possible reason is that the level of economic development of the eastern region is high, and the factors that can used for agricultural production are relatively well developed. In addition, the eastern region has a unique location advantage, and the congenital conditions of agricultural production are better than in other regions. The regression coefficient of the policy variable in the central region is negative and non-significant, indicating that for the central region, the CFSDC has actually inhibited agricultural development. A possible reason is that, on the one hand, the central region still dominated by the primary industry and the secondary industry. However, compared with the eastern region, the agricultural production technology and agricultural management experience of the cities in the central region are relatively low. To meet the higher assessment requirements of the food safety demonstration cities, an excessive amount of local intervention in agricultural development is needed, which affects the normal order of agricultural production. On the other hand, as the central region is adjacent to the eastern region, it plays an important role for the industrial transfer of the eastern region. Urban development focuses on the development of the secondary industry, with relatively little emphasis on effectively improving agricultural production efficiency. This may affect the effect of the policy on the creation of food safety model cities. Although the regression coefficient of the policy variable in the western region is positive, it is not significant. A possible reason is that the policy effect of the food safety demonstration city cannot be fully realized because of the poor innate conditions of agricultural production in the western region and the relative lack of value agricultural production resources.

#### 4.5.3. Heterogeneity Analysis of Urban Administrative Level

The degree of preferential policies enjoyed by cities has been shown to be inextricably linked to their administrative rank. Administrative level is an important dimension for determining the absorptive capacity of cities, and cities with a high administrative level enjoy preferential access to resources compared to cities with low or medium administrative levels [49]. Therefore, cities with different administrative levels may also perform differently in the policy pilot activity of creating a model food safety city. Based on this, the 277 cities are divided into two samples: high-administrative-level cities and low- and medium-administrative-level cities. High-administrative-level cities include all municipalities and provincial capitals, and the first-tier cities that are not provincial capitals are included in the high-administrative-level cities sample in reference to the “2021 City Commercial Charm Rankings”. The resulting high-administrative-level city sample includes 36 cities, and the medium- and low-administrative-level city sample contains 241 cities.

Models 4 and 5 in Table 7 report the regression coefficients of the impact of urban administrative level heterogeneity on agricultural development. The regression coefficient of the policy variable for the high-administrative-level city sample is positive. This indicates that for high-administrative-level cities, the creation of food safety model cities significantly contributes to agricultural development. In addition, the absolute value of regression coefficients for policy variables is larger for high-administrative-level cities compared to the baseline regression results. This difference suggests that high-administrative-level cities enjoy priority access to resources and consequently develop quicker than low- and medium-administrative-level cities. For high-administrative-level cities, the creation of food safety demonstration cities is the “icing on the cake”. The regression coefficient of 0.0021 on the policy variable for the low and medium ranked cities is not significant, suggesting that for low- and medium-administrative-level cities, the pilot policy of creating food safety demonstration cities promotes agricultural development. However, the policy effect is not significant and the cities’ agricultural development must adjust its current resource allocation pattern and agricultural production pattern and improve efficiency.

## 5. Conclusions and Implications

### 5.1. Conclusions

Based on panel data of 277 prefecture-level cities in China from 2011 to 2019, the impact of the creation of food safety demonstration cities (CFSDC) on agricultural development and its mechanism of action are analyzed using a multi-period DID model. The findings are summarized in the following: (1) The CFSDC significantly promotes urban agricultural development. This conclusion still holds under a series of robustness tests such as PSM-DID test and placebo test. (2) The CFSDC promotes urban agricultural development through two channels: accelerating technological innovation and accelerating industrial structure adjustment. (3) The impact the CFSDC has on agricultural development is clearly heterogeneous. The policy effect diminishes as cities expand in size, and the policy effect is most pronounced in the eastern region. The CFSDC is the “icing on the cake” for high-administrative-level cities, and the policy effect is most pronounced in the eastern region.

### 5.2. Policy Implications

First, decision makers should pay attention to the impact of pilot policies on agricultural development. This paper shows that the policy of creating food safety demonstration cities has both improved food safety and promoted agricultural development across China. Therefore, it is important to further enhance the promotion of food safety demonstration cities and related pilot policies. Additionally, the organic combination of pilot policies and agricultural development should be promoted, to achieve the goal of “evaluation for construction”.

Second, importance should be attached to the combination of the pilot policy of “adapting measures to local conditions” and “adapting measures to the time”. This paper shows that the impact of food safety policies on agricultural development can be heterogeneous depending on the size, location, and level of the city. Therefore, when implementing this pilot policy, the objective reality of agricultural development and economic and social development in the region should be integrated to promote the rational allocation of resources in different regions, departments, and time points, to realize the optimal utilization of resources.

Third, attention should be directed to the intermediary effect of technological innovation and industrial structure adjustment. This paper shows that the CFSDC indirectly promotes urban agricultural development through technological innovation and industrial structure adjustment. Therefore, a high-level agricultural talent exchange and technology R&D transformation should be built on a wider and deeper scale to promote urban innovation capabilities. Moreover, the industrial structure of the region should be rationally deployed, financial support for modern agriculture should be increased, and the secondary and tertiary industries should be promoted to feed back into the primary industry.

## Figures and Tables

**Table 1:** Baseline regression results.

Variable	(1)	(2)	(3)	(4)	(5)	(6)
DID	0.0094 ***	0.0075 ***	0.0076 ***	0.0076 ***	0.0077 ***	0.0077 ***
	(3.5597)	(2.7982)	(3.0019)	(3.0638)	(3.1336)	(3.0442)
CLA		0.0170 ***	0.0098	0.0091	0.0097	0.0079
		(2.9627)	(1.3309)	(0.6249)	(0.6655)	(0.5187)
UAM			0.0063	0.0062	0.0062	0.0070
			(1.5345)	(1.5254)	(1.5338)	(1.5962)
UAP				0.0008	0.0001	0.0021
				(0.0657)	(0.0060)	(0.1612)
EDL					0.0031	0.0028
					(0.7696)	(0.7098)
DOO						−0.0030 ***
						(−3.1815)
Year Fixed Effect	Yes	Yes	Yes	Yes	Yes	Yes
City Fixed Effect	Yes	Yes	Yes	Yes	Yes	Yes
Obs	2493	2493	2493	2493	2493	2493
R^2^	0.488	0.500	0.502	0.502	0.503	0.508

Note: *, **, and *** indicate significance at the 10%, 5%, and 1% significance levels, respectively, with t values in parentheses. The following tables are the same.

**Table 2:** PSM-DID test results.

Variable	Radius Matching	Kernel Matching	Nearest Neighbor Matching
DID	0.0064 *	0.0064 *	0.0067 *
	(1.8727)	(1.8325)	(1.9392)
Control Variable	Yes	Yes	Yes
Year Fixed Effect	Yes	Yes	Yes
City Fixed Effect	Yes	Yes	Yes
Obs	2289	2284	2273
R^2^	0.5157	0.5158	0.5168

**Table 3:** Further robustness test results.

Variable	Lagging the Control Variable	Abbreviated Processing	Adjusting the Sample Interval	Replacing the Explained Variable
DID	0.0094 ***	0.0074 ***	0.0054 **	0.0077 ***
	(3.4978)	(2.7447)	(2.2252)	(3.0442)
Control Variable	Yes	Yes	Yes	Yes
Year Fixed Effect	Yes	Yes	Yes	Yes
City Fixed Effect	Yes	Yes	Yes	Yes
Obs	2216	2493	1939	2493
R^2^	0.4980	0.5060	0.5822	0.5080

**Table 4:** Test results of the mediating effect of technological innovation.

Variable	Agricultural Development	Technological Innovation	Agricultural Development	Agricultural Development
DID	0.0077 ***	1.3136 ***		0.0055 **
	(3.0442)	(3.6639)		(2.1598)
Technological Innovation			0.0019 ***	0.0017 ***
			(5.5944)	(5.5556)
Control Variable	Yes	Yes	Yes	Yes
Year Fixed Effect	Yes	Yes	Yes	Yes
City Fixed Effect	Yes	Yes	Yes	Yes
Obs	2493	2493	2493	2493
R^2^	0.508	0.211	0.511	0.513

**Table 5:** Test results of the mediating effect of industrial structure adjustment.

Variable	Agricultural Development	Industrial Structure Adjustment	Agricultural Development	Agricultural Development
DID	0.0077 ***	0.0514 ***		0.0064 ***
	(3.0442)	(3.7042)		(2.6717)
Industrial Structure Adjustment			0.0243 ***	0.0237 ***
			(4.9365)	(4.8750)
Control Variable	Yes	Yes	Yes	Yes
Year Fixed Effect	Yes	Yes	Yes	Yes
City Fixed Effect	Yes	Yes	Yes	Yes
Obs	2493	2493	2493	2493
R^2^	0.508	0.312	0.521	0.523

**Table 6:** Estimation results of city size heterogeneity.

Variable	Megacities	Large Cities	Medium and Small Cities
DID*TYPE	0.0052 **	0.0077 *	0.0165 ***
	(2.0431)	(1.9255)	(6.6103)
Control Variable	Yes	Yes	Yes
Year Fixed Effect	Yes	Yes	Yes
City Fixed Effect	Yes	Yes	Yes
Obs	2493	2493	2493
R^2^	0.506	0.506	0.506

**Table 7:** Estimated results of urban location and urban administrative level heterogeneity.

Variable	Eastern Region	Central Region	Western Region	High-Level Cities	Low- and Middle-Level Cities
DID*TYPE	0.0125 ***	−0.0028	0.0070	0.0112 ***	0.0021
	(5.6983)	(−0.5940)	(1.6222)	(5.2352)	(0.5086)
Control Variable	Yes	Yes	Yes	Yes	Yes
Year Fixed Effect	Yes	Yes	Yes	Yes	Yes
City Fixed Effect	Yes	Yes	Yes	Yes	Yes
Obs	2493	2493	2493	2493	2493
R^2^	0.509	0.505	0.506	0.509	0.505

## Data Availability

Not applicable.

## References

[1] Ding Z., Zhang R., Kan Z. (2015). Quality and safety inspection of food and agricultural products by LabVIEW IMAQ vision. Food Anal. Methods.

[2] Darby M.R., Karni E. (1973). Free competition and the optimal amount of fraud. J. Law Econ..

[3] Chen M., Wang Y., Yin S., Hu W., Han F. (2019). Chinese consumer trust and preferences for organic labels from different regions: Evidence from real choice experiment. Br. Food J..

[4] Crandall P.G., Mauromoustakos A., O’Bryan C.A., Thompson K.C., Yiannas F., Bridges K., Francois C. (2017). Impact of the global food safety initiative on food safety worldwide: Statistical analysis of a survey of international food processors. J. Food Prot..

[5] Unnevehr L.J. (2022). Addressing food safety challenges in rapidly developing food systems. Agric. Econ..

[6] Tang H., Liu Y., Huang G. (2019). Current status and development strategy for community-supported agriculture (CSA) in China. Sustainability.

[7] Zhang Y., Diao X. (2020). The changing role of agriculture with economic structural change—The case of China. China Econ. Rev..

[8] Li H., He J., Bharucha Z.P., Lal R., Pretty J. (2016). Improving China’s food and environmental security with conservation agriculture. Int. J. Agric. Sustain..

[9] Scott S., Si Z., Schumilas T., Chen A. (2014). Contradictions in state- and civil society-driven developments in China’s ecological agriculture sector. Food Policy.

[10] Li W., Clark B., Taylor J.A., Kendall H., Jones G., Li Z., Jin S., Zhao C., Yang G., Shuai C. (2020). A hybrid modelling approach to understanding adoption of precision agriculture technologies in Chinese cropping systems. Comput. Electron. Agric..

[11] Tscharntke T., Clough Y., Wanger T.C., Jackson L., Motzke I., Perfecto I., Vandermeer J., Whitbread A. (2012). Global food security, biodiversity conservation and the future of agricultural intensification. Biol. Conserv..

[12] Madanayake N.H., Hossain A., Adassooriya N.M. (2021). Nanobiotechnology for agricultural sustainability, and food and environmental safety. Qual. Assur. Saf. Crops Foods.

[13] Baer W.S., Johnson L.L., Merrow E.W. (1977). Government-sponsored demonstrations of new technologies: A well-developed technology, user participation, and risk-sharing lead to more rapid commercial adoption. Science.

[14] Macey S.M., Brown M.A. (1990). Demonstrations as a policy instrument with energy technology examples. Knowledge.

[15] Erjavec E., Volk T., Rednak M., Rac I., Zagorc B., Moljk B., Zgajnar J. (2017). Interactions between European agricultural policy and climate change: A slovenian case study. Clim. Policy.

[16] Lankoski J., Thiem A. (2020). Linkages between agricultural policies, productivity and environmental sustainability. Ecol. Econ..

[17] Schmidt N.M. (2020). Late bloomer? Agricultural policy integration and coordination patterns in climate policies. J. Eur. Public Policy.

[18] Ecker O., Hatzenbuehler P.L. (2022). Food consumption-production response to agricultural policy and macroeconomic change in Nigeria. Appl. Econ. Perspect. Policy.

[19] Conning J.H., Robinson J.A. (2007). Property rights and the political organization of agriculture. J. Dev. Econ..

[20] Jacoby H., Minten B. (2006). Land Titles, Investment, and Agricultural Productivity In Madagascar: A Poverty and Social Impact Analysis.

[21] Kendall H., Clark B., Li W., Jin S., Jones G.D., Chen J., Taylor J., Li Z., Frewer L.J. (2022). Precision agriculture technology adoption: A qualitative study of small-scale commercial “family farms” located in the North China Plain. Precis. Agric..

[22] Ehlers M.-H., Huber R., Finger R. (2021). Agricultural policy in the era of digitalisation. Food Policy.

[23] Azadi H., Ghanian M., Ghoochani O.M., Rafiaani P., Taning C.N.T., Hajivand R.Y., Dogot T. (2015). Genetically modified crops: Towards agricultural growth, agricultural development, or agricultural sustainability?. Food Rev. Int..

[24] Ramirez-Hernandez A., Galagarza O.A., Alvarez Rodriguez M.V., Pachari Vera E., Valdez Ortiz M.d.C., Deering A.J., Oliver H.F. (2020). Food safety in Peru: A review of fresh produce production and challenges in the public health system. Compr. Rev. Food Sci. Food Saf..

[25] Sikandar F., Erokhin V., Wang H., Rehman S., Ivolga A. (2021). The impact of foreign capital inflows on agriculture development and poverty reduction: Panel data analysis for developing countries. Sustainability.

[26] Poulton C. (2014). Democratisation and the political incentives for agricultural policy in Africa. Dev. Policy Rev..

[27] Mockshell J., Birner R. (2015). Donors and domestic policy makers: Two worlds in agricultural policy-making?. Food Policy.

[28] Challinor A.J., Watson J., Lobell D.B., Howden S.M., Smith D.R., Chhetri N. (2014). A meta-analysis of crop yield under climate change and adaptation. Nat. Clim. Change.

[29] Laurance W.F., Sayer J., Cassman K.G. (2014). Agricultural expansion and its impacts on tropical nature. Trends Ecol. Evol..

[30] King T., Cole M., Farber J.M., Eisenbrand G., Zabaras D., Fox E.M., Hill J.P. (2017). Food safety for food security: Relationship between global megatrends and developments in food safety. Trends Food Sci. Technol..

[31] Pei X., Li N., Guo Y., Liu X., Yan L., Li Y., Yang S., Hu J., Zhu J., Yang D. (2015). Microbiological food safety surveillance in China. Int. J. Environ. Res. Public. Health.

[32] Chen T., Ding K., Yu Z., Li G., Dong Y.I. (2021). Smart supervision for food safety in food service establishments in China: Challenges and solutions. J. Food Prot..

[33] Yu B. (2020). Industrial structure, technological innovation, and total-factor energy efficiency in China. Environ. Sci. Pollut. Res..

[34] Smith H.E., Sallu S.M., Whitfield S., Gaworek-Michalczenia M.F., Recha J.W., Sayula G.J., Mziray S. (2021). Innovation systems and affordances in climate smart agriculture. J. Rural Stud..

[35] Li X., Guo D., Feng C. (2022). The carbon emissions trading policy of China: Does it really promote the enterprises’ green technology innovations?. Int. J. Environ. Res. Public. Health.

[36] Guo H., Wang Y., Yang Z., Zhang Q. (2022). Spatial-temporal pattern envolution of agricultural economic development level in Sichuan Province-based on panel data from 2006 to 2019. Chin. J. Agric. Res. Reg. Plan..

[37] Ji X., Xu J., Zhang H. (2023). Environmental effects of rural E-commerce: A case study of chemical fertilizer reduction in China. J. Environ. Manag..

[38] Tu S., Xu F. (2020). Agricultural economic growth and agricultural environmental pollution-analysis based on spatial effect. Rural Econ..

[39] Zhou F., Wang X. (2022). The carbon emissions trading scheme and green technology innovation in China: A new structural economics perspective. Econ. Anal. Policy.

[40] Wang Q., Hu A., Tian Z. (2022). Digital transformation and electricity consumption: Evidence from the Broadband China Pilot Policy. Energy Econ..

[41] Zhang H., Huang L., Zhu Y., He X. (2021). Does low-carbon city construction improve total factor productivity? Evidence from a quasi-natural experiment in China. Int. J. Environ. Res. Public. Health.

[42] Beck T., Levine R., Levkov A. (2010). Big Bad Banks? The Winners and Losers from Bank Deregulation in the United States. J. Financ..

[43] Pan X., Pu C., Yuan S., Xu H. (2022). Effect of Chinese pilots carbon emission trading scheme on enterprises’ total factor productivity: The moderating role of government participation and carbon trading market efficiency. J. Environ. Manage..

[44] Zhang S., Wu Z., He Y., Hao Y. (2022). How does the green credit policy affect the technological innovation of enterprises? Evidence from China. Energy Econ..

[45] Baron R.M., Kenny D.A. (1986). The moderator–mediator variable distinction in social psychological research: Conceptual, strategic, and statistical considerations. J. Pers. Soc. Psychol..

[46] Zhang H., Sun C., Huang L., Si H. (2021). Does government intervention ensure food safety? Evidence from China. Int. J. Environ. Res. Public. Health.

[47] Chong Z., Qin C., Ye X. (2017). Environmental regulation and industrial structure change in China: Integrating spatial and social network analysis. Sustainability.

[48] Zhu S., Li D., Feng H., Gu T., Hewage K., Sadiq R. (2020). Smart city and resilient city: Differences and connections. Wires Data Min. Knowl. Discov..

[49] Zheng H., Többen J., Dietzenbacher E., Moran D., Meng J., Wang D., Guan D. (2021). Entropy-based Chinese city-level MRIO table framework. Econ. Syst. Res..

